# Validation of a Janus role of methotrexate-based PEGylated chitosan nanoparticles in vitro

**DOI:** 10.1186/1556-276X-9-363

**Published:** 2014-07-23

**Authors:** Fanghong Luo, Yang Li, Mengmeng Jia, Fei Cui, Hongjie Wu, Fei Yu, Jinyan Lin, Xiangrui Yang, Zhenqing Hou, Qiqing Zhang

**Affiliations:** 1 Department of Chemistry, College of Chemistry and Chemical Engineering, Xiamen University, Xiamen 361005, China; 2 Department of Biomaterials and Research Center of Biochemical Engineering, College of Materials, Xiamen University, Xiamen 361005, China; 3 Department of Pharmacy, School of Pharmaceutical Sciences, Xiamen University, Xiamen 361002, China; 4 Cancer Research Center, Medical College, Xiamen University, Xiamen 361005, China; 5 Institute of Biomedical Engineering, Chinese Academy of Medical Science and Peking Union Medical College, Tianjin 300192, China

**Keywords:** Methotrexate, Chitosan, Drug delivery system, Tumor, Nanoparticles

## Abstract

Recently, methotrexate (MTX) has been used to target to folate (FA) receptor-overexpressing cancer cells for targeted drug delivery. However, the systematic evaluation of MTX as a Janus-like agent has not been reported before. Here, we explored the validity of using MTX playing an early-phase cancer-specific targeting ligand cooperated with a late-phase therapeutic anticancer agent based on the PEGylated chitosan (CS) nanoparticles (NPs) as drug carriers. Some advantages of these nanoscaled drug delivery systems are as follows: (1) the NPs can ensure minimal premature release of MTX at off-target site to reduce the side effects to normal tissue; (2) MTX can function as a targeting ligand at target site prior to cellular uptake; and (3) once internalized by the target cell, the NPs can function as a prodrug formulation, releasing biologically active MTX inside the cells. The (MTX + PEG)-CS-NPs presented a sustained/proteases-mediated drug release. More importantly, compared with the PEG-CS-NPs and (FA + PEG)-CS-NPs, the (MTX + PEG)-CS-NPs showed a greater cellular uptake. Furthermore, the (MTX + PEG)-CS-NPs demonstrated a superior cytotoxicity compare to the free MTX. Our findings therefore validated that the MTX-loaded PEGylated CS-NPs can simultaneously target and treat FA receptor-overexpressing cancer cells.

## Background

Nanotechnology is a rapidly advancing and key field of drug delivery. A great variety of nanoparticle (NP)-based therapeutic products have entered clinical development or been approved for clinical use [1]. As an excellent biocompatible and biodegradable nanomaterial with low toxicity and immunogenicity, chitosan (CS)-based nanocarriers presented great advantages for drug, protein, and gene delivery in therapeutics [2-5]. However, most CS-based nanocarriers were easily sequestered by macrophages in the mononuclear phagocyte system (MPS) after intravenous administration. To avoid the rapid clearance of the CS-NPs during circulation, PEGylation can be used to improve the physiological stability, reduce the opsonization, and increase the possibility reaching the tumor by the enhanced permeation and retention (EPR) effect (40 to 400 nm) [6-8].

Despite these advantages of the passive targeting, the main obstacle encountered with the clinical use of the PEGylated CS-NPs is how to facilitate their internalization in the target cells while reducing the unintended side effects. One strategy is the further functionalization of the PEGylated CS-NPs with active targeting agents. For instance, some ligands or antibodies could specifically recognize the receptors or antigens on the surface of various cancer cells [9]. Notably, the exploitation of folate (FA) receptor for targeted drug delivery has long been persued. FA receptors were overexpressed in a wide variety of cancer cells, including ovarian, lung, breast, kidney, and brain cancer cells, but its level is very low in normal cells [10,11]. Previously, we synthesized the CS-NPs by the combination of ionic gelation and chemical cross-linking method and prepared the (FA + PEG)-CS-NPs by dual-conjugation with mPEG-SPA and FA [12]; the enhanced cellular uptake and tumor accumulation also inspired our motivation of adopting the CS-NPs as drug carriers to continue our studies for an extensively used anticancer drug methotrexate (MTX).

**Figure 1:**
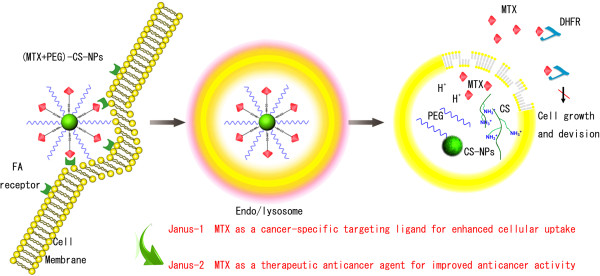
**Mechanism of Janus role of the (MTX + PEG)-CS-NPs.** Once intravenously administrated, it was anticipated that the (MTX + PEG)-CS-NPs were accumulated at the tumor site by the EPR effect. Prior to the cellular take, the (MTX + PEG)-CS-NPs were served similarly as a targeted drug delivery system, in which MTX can function as a targeting moiety and selectively transport the NPs to the target cells. Once internalized into the target cells, the (MTX + PEG)-CS-NPs were served similarly as a prodrug system, in which MTX would be released inside the cells and function as a therapeutic anticancer agent. Additionally, the protease-mediated drug release could ensure that MTX timely change its role from targeting (via FA receptor-mediated endocytosis) to anticancer (inhibit DHFR activity and stop FA cycle). This work systematically revealed the unanticipated targeting coordinated with anticancer efficiency of Janus-like MTX in vitro.

MTX, as an analogue of FA for high structural similarity, can enter cells by reduced FA carrier, proton-coupled FA transporter, or membrane-associated FA receptor [13-15]. MTX could inhibit dihydrofolate reductase (DHFR) activity and stop FA cycle, and in turn inhibit the DNA synthesis and cell proliferation, and finally drives cells to death [16-18]. Recently, MTX has been developed to target to FA receptor-overexpressing cancer cells in vitro [19-21]. These encouraged the vision and enhanced the scope of Janus-like MTX as an early-phase cancer-specific targeting ligand coordinated with a late-phase therapeutic anticancer agent with promising potential in vitro and in vivo. Particularly, Janus role of MTX as a promising candidate has attracted an increasing interest and may provide a new concept for drug delivery and cancer therapy [22-25]. Validation is also a crucial step in the drug discovery process [26,27]. To prove the validity and investigate the efficiency of the Janus role on the nanoscaled drug delivery systems, our present work is greatly enthused by the Janus-like MTX and we used the PEGylated CS-NPs to develop the Janus-like (MTX + PEG)-CS-NPs. Mechanisms of their targeting and anticancer dual effect were schematically illustrated in Figure 1.

## Methods

### Materials

All chemical reagents were of analytical grade and used without further purification unless otherwise stated. Chitosan (CS, Mw = 70,000 Da, 95% degree of deacetylation) was purchased from Zhejiang Aoxing Biotechnology Co., Ltd. (Zhengjiang, China). 1-Ethyl-3-(3-dimethylaminopropyl) carbodiimide (EDC), *N*-hydroxysuccinimide (NHS), and crude proteases from bovine pancreas were purchased from Sigma Chemical Corp (St. Louis, MO, USA). Folate (FA) and methotrexate (MTX) were purchased from Bio Basic Inc. (Markham, Ontario, Canada). *N*-Succinimidyl ester of methoxypolyethylene glycol propionic acid (mPEG-SPA, Mw = 2,000 Da) was purchased from Jiaxing Biomatrix Inc. (Zhengjiang, China). A dialysis bag (Mw = 8,000 to 14,000 Da) was ordered from Greenbird Inc. (Shanghai, China). A Spectra/Por dialysis membrane (Mw = 6,000 to 8,000 Da) was purchased from Spectrum Laboratories (Rancho Domingues, CA, USA). Deionized (DI) water was used throughout. Fetal bovine serum (FBS) was purchased from Gibco Life Technologies (AG, Zug, Switzerland). Trypsin-EDTA (0.25%) and penicillin-streptomycin solution was from Invitrogen. All solvents used in this study were high-performance liquid chromatography (HPLC) grade. HeLa cells and MC 3 T3-E1 cells were provided by American Type Culture Collection (ATCC, Manassas, VA, USA).

### Preparation of the (MTX + PEG)-CS-NPs

Firstly, the CS-NPs were prepared by the ionic gelation combined with chemical cross-linking method according to our previous work [12]. Secondly, mPEG-SPA (50 mg) was added into the CS-NPs suspensions (5 mL, 10 mg/mL) accompanied by vigorous stirring for 4 h. The prepared PEG-CS-NPs were dialyzed against DI water to remove excess of mPEG-SPA using a dialysis bag (Mw = 8,000 to 14,000 Da) and lyophilized for 24 h. Lastly, MTX (5 mg), EDC (8 mg), and NHS (5 mg) were dissolved in 5 mL of PBS (pH = 7.4). The pH was adjusted to 6.0 by the addition of 0.2 M HCl. The mixture was allowed to react for 30 min and added dropwise to the PEG-CS-NPs suspension (5 mL, 10 mg/mL). The pH was adjusted to 8.0 with 0.2 M NaOH. The reaction was allowed to occur at room temperature for 48 h. Following MTX conjugation, the (MTX + PEG)-CS-NPs NPs were centrifuged at 20,000 rpm for 30 min at 4°C, washed with PBS/DI water, and lyophilized for 24 h. All of the supernatants were collected for further indirect calculation of the drug-loading content. The (FA + PEG)-CS-NPs were prepared by the same method.

### Physicochemical characterization of (MTX + PEG)-CS-NPs

Fourier transform infrared spectroscopy (FTIR) spectrum analysis of (MTX + PEG)-CS-NPs was performed using a NicoletAVTAR36 FTIR Spectrometer (Thermo Scientific, Salt Lake City, UT, USA). For comparison, The CS-NPs, PEG, PEG-CS-NPs, and MTX were used as controls.

Average particle size and polydispersity index (PDI) were determined by dynamic light scattering (DLS) using a Malvern Zetasizer Nano-ZS (Malvern Instruments, Worcestershire, UK). Zeta potential was evaluated by electrophoretic light scattering (ELS) with Zetaplus (Brookhaven Instruments Corporation, Holtsville, NY, USA). Particle size was evaluated by intensity distribution. Atomic force microscopy (AFM) study was performed on a Nanoscope Multimode atomic force microscope (Veeco Instruments Inc., New York, USA). Transmission electron microscopy (TEM) image was obtained on a JEM 2100 transmission electron microscope (JEOL, Tokyo, Japan).

The amount of drug in the supernatant was assayed using a high-performance liquid chromatography (Waters Associates, Milford, MA, USA) system with the following conditions: stationary phase, Hypersill ODS column (250 mm × 4.6 mm, 5 μm); mobile phase, potassium dihydrogen phosphate buffer (pH 4.5)-acetonitrile (88:12); elution flow rate, 1 mL/min; and detection wavelength, 303 nm. The drug-loading content was calculated according to the previous report [12].

### In vitro stability tests

PBS stability test against ionic strength and plasma stability test against protein adsorption were evaluated immediately after preparation and subsequently at regular intervals. Briefly, 5 mg of the lyophilized (MTX + PEG)-CS-NPs were suspended in PBS (pH 7.4) or 10% (*v*/*v*) plasma/heparin in PBS and stored at 37°C for 120 h. The particle size was determined at 0, 24, 48, 72, 96, and 120 h, respectively.

### In vitro drug release

In vitro release of MTX from the (MTX + PEG)-CS-NPs was evaluated by a dialysis method. The lyophilized (MTX + PEG)-CS-NPs suspended in 10% plasma (with or without the presence of crude proteases) were added into a dialysis bag (Mw = 6,000 to 8,000 Da) and immersed into the release medium at 37°C with agitation. At the predesigned time points, 2 mL of the release medium was completely withdrawn and subsequently replaced with the same volume of fresh PBS. For comparison, in vitro release of the free MTX was evaluated as a control.

### Cell culture

HeLa cells were cultured in FA-deficient Dulbecco’s Modified Eagle’s Medium (DMEM) supplemented with 10% fetal bovine serum (FBS) and 1% penicillin-streptomycin. MC 3 T3-E1 cells were cultured in Minimum Essential Medium, Alpha Modified (α-MEM), under similar conditions. The two cell lines have different levels of FA receptor expression. In particular, HeLa cells (cancer cells) are FA receptor positive, and MC 3 T3-E1 cells (normal cells) are FA receptor negative. All of the cells were cultivated in a 5% CO_2_-humidified atmosphere at 37°C.

### In vitro cellular uptake

To qualitatively investigate the cellular uptake of the PEG-CS-NPs, (FA + PEG)-CS-NPs or (MTX + PEG)-CS-NPs, fluorescein isothiocyanate (FITC) was conjugated to different formulations to prepare the FITC-PEG-CS-NPs, FITC-(FA + PEG)-CS-NPs or FITC-(MTX + PEG)-CS-NPs. HeLa cells were seeded at a density of 8 × 10^4^ cells per well into 6-well plates with their specific cell culture medium. The cells were incubated at 37°C and 5% CO_2_ for 24 h. One hundred microliters of the FITC-PEG-CS-NPs, FITC-(FA + PEG)-CS-NPs, or FITC-(MTX + PEG)-CS-NPs was added to HeLa cells at the equivalent concentration of FITC and incubated further for 6 h. HeLa cells were washed with PBS and stained with Hoechst 33258. Then, HeLa cells were washed with PBS and fixed with 4% formaldehyde. The cells were observed using a Leica TCS SP5 laser confocal scanning microscopy (Leica Microsystems, Mannheim, Germany).

To quantitatively investigate the internalization of the FITC-labeled (MTX + PEG)-CS-NPs, (FA + PEG)-CS-NPs or PEG-CS-NPs, HeLa cells were incubated in 6-well plates at a density of 2 × 10^5^ cells/mL and allowed to grow for 24 h. The FITC-(MTX + PEG)-CS-NPs, FITC-(FA + PEG)-CS-NPs, or FITC-PEG-CS-NPs at the equivalent concentration of FITC were then added to each well. After incubation for 4 h, the cells were washed with cold PBS twice, harvested by 0.25% (*w*/*v*) trypsin/0.03% (*w*/*v*) EDTA, centrifuged at 1,000 rpm for 5 min at 4°C and resuspended in PBS for the analysis by a Coulter EPICS XL Flow Cytometer (Beckman Coulter Inc., Brea, CA, USA).

### In vitro cell viability studies

Cytotoxicity of the PEG-CS-NPs, (FA + PEG)-CS-NPs, (MTX + PEG)-CS-NPs, and free MTX were evaluated by MTT assay. HeLa cells (cancer cells) or MC 3 T3-E1 cells (normal cells) were seeded at a density of 3 × 10^3^ cells per well into 96-well plates with their specific cell culture medium. The cells were incubated at 37°C in humidified atmosphere containing 5% CO_2_ for 24 h. The medium was then replaced with fresh medium, and different formulations were added to incubate with the cells. After 24 h of incubation, the medium was removed; each well was rinsed with PBS; and 20 μL of MTT solution was added followed by incubation for 4 h. Then, the metabolized product MTT formazan was dissolved by adding 200 μL of DMSO to each well. Finally, the plate was shaken for 20 min, and the absorbance of the formazan product was measured at 570 nm in a microplate reader (Bio-Rad, Model 680, Bio-Rad Laboratories, Richmond, CA, USA).

### Subcellular localization

To further understand the mechanisms of in vitro cell viability studies, we investigated the subcellular localization using a laser confocal scanning microscopy. After the predesigned incubation times with the FITC-labeled (MTX + PEG)-CS-NPs, HeLa cells were washed with PBS and stained with LysoTracker Red following the manufacturer’s instructions. The cells were then washed with PBS, fixed with 4% formaldehyde for 15 min and observed by a laser confocal scanning microscopy.

## Results and discussion

### Preparation of the (MTX + PEG)-CS-NPs

**Figure 2:**
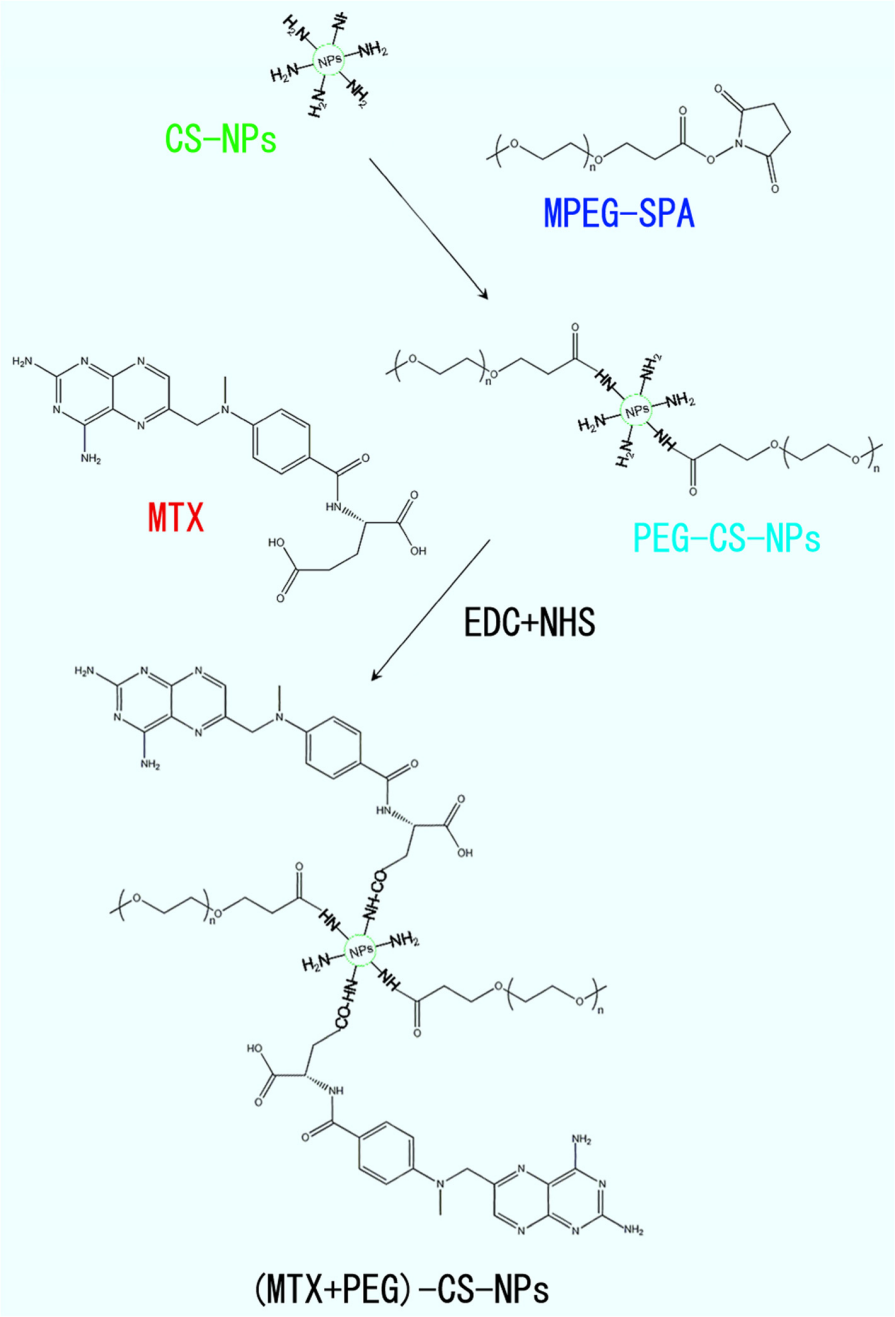
Synthetic scheme of the (MTX + PEG)-CS-NPs.

We used a two-step procedure for the preparation of the (MTX + PEG)-CS-NPs based on the CS-NPs (Figure 2). Firstly, the succinimidyl groups of mPEG-SPA were conjugated to the amino groups of the CS-NPs, as the PEG-CS-NPs with methoxy surface groups were ideal for drug delivery [28]. Subsequently, the γ-carboxyl groups within MTX were conjugated to the residual amino groups of PEG-CS-NPs via carbodiimide chemistry [19]. As is reported, FA retained a high affinity for FA receptor even when conjugated with a wide variety of molecules, thus making it a potential useful tumor-targeting ligand [10].Thus, it is expected that the conjugation of the MTX molecule with the PEGylated CS-NPs could not only preserve its accessibility to the FA receptor site to exert the targeting effect, but concomitantly avoid its premature release to reduce the side effects of chemotherapy.

### Physicochemical characterization of the (MTX + PEG)-CS-NPs

**Figure 3:**
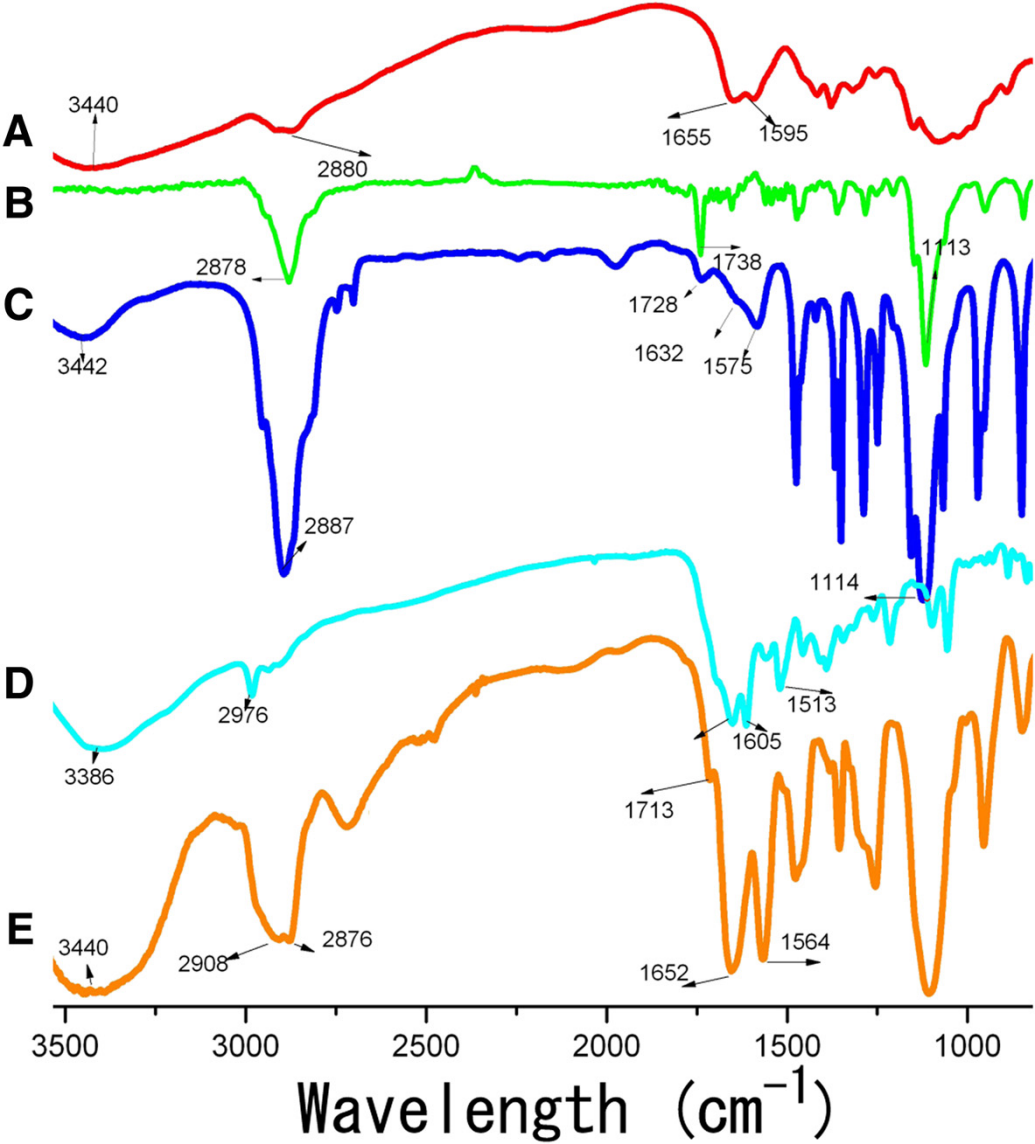
FTIR spectra of the (A) CS-NPs, (B) PEG, (C) PEG-CS-NPs, (D) MTX, and (E) (MTX + PEG)-CS-NPs.

FTIR analysis. The comparative FTIR spectra of all kinds of NPs were shown in Figure 3. The CS-NPs showed a broad band at 3,440 cm^-1^, which was assigned to the superposition of N-H and O-H stretching vibration of the polymer backbone of the CS-NPs. Following the modification of mPEG-SPA, an intensity increase was observed in the alkyl C-H stretching vibration at 2,887 cm^-1^. The peaks at 1,728 and 1,114 cm^-1^ indicated the C = O and C-O-C stretching vibration native to the structure of mPEG-SPA, respectively. These results testified to the successful PEGylation. After the further modification of MTX, the peak at 1,713 cm^-1^ indicated the generation of new C = O stretching vibration, more importantly, the appearance of the peaks at 1,652 and 1,564 cm^-1^ were indicative of the introduction of a greater conjugated system; in other words, the results suggested that the interaction between PEG-CS-NPs and MTX was at the level of a new amide bond.

**Figure 4:**
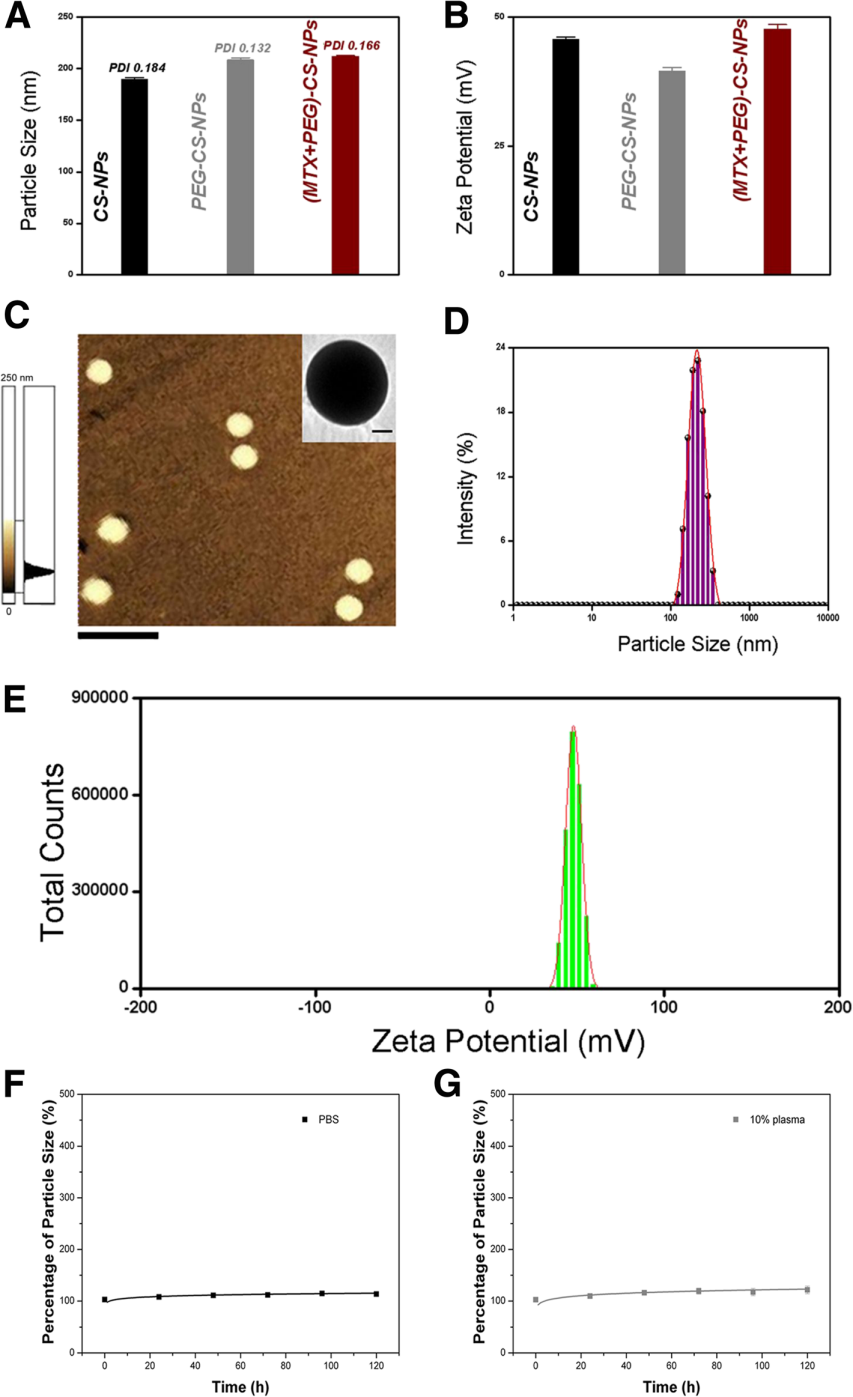
**Physicochemical characterization of the (MTX + PEG)-CS-NPs. (A)** Particle size of the CS-NPs, PEG-CS-NPs, and (MTX + PEG)-CS-NPs (mean ± SD, *n* = 3). **(B)** Zeta potential of the CS-NPs, PEG-CS-NPs, and (MTX + PEG)-CS-NPs (mean ± SD, *n* = 3). **(C)** AFM image of the (MTX + PEG)-CS-NPs. Scale bars = 500 nm. Inset: TEM image of the (MTX + PEG)-CS-NPs. Scale bars = 50 nm. **(D)** Particle size distribution of the (MTX + PEG)-CS-NPs. **(E)** Zeta potential distribution of the (MTX + PEG)-CS-NPs. **(F)** In vitro stability tests of the (MTX + PEG)-CS-NPs in PBS (mean ± SD, *n* = 3). **(G)** In vitro stability tests of the (MTX + PEG)-CS-NPs in 10% plasma in PBS (mean ± SD, *n* = 3).

Particle size, PDI, zeta potential, and morphology. Surface biofunctionalization was accompanied by the changes in particle size (Figure 4A) and zeta potential (Figure 4B) of the NPs. After PEGylation and MTX modification, the particle size increased from 190.1 to 213.4 nm, and the zeta potential decreased from 45.7 to 39.6 mV, and then increased to 47.9 mV. Particle size of approximately 200 nm was suited for the prolonged circulation because they were big enough to avoid the rapid uptake by the RES but small enough to avoid the rapid renal clearance [7,29]. The best EPR effect for a rigid particle is achieved for particle size <400 nm [6,30]. Surface charge is an important indication for the stability of the nanoscaled drug delivery system in the physiological environment. The electrostatic repulsion among the NPs with the same type of surface charge would confer stability [31,32]. The (MTX + PEG)-CS-NPs presented a spherical shape (Figure 4C), a nanoscaled particle size (Figure 4D), a narrow particle size distribution (Figure 4D), a high zeta potential (Figure 4E), a moderate drug-loading content (7.23 ± 0.11%, discussed below), and a good physiological stability (see Figure 4F,G, discussed below), indicating that they were effective therapeutic drug delivery systems [1].

Drug-loading content. CS-NPs possessing peripheral amino groups provided us great opportunities to easy surface biofunctionalization. In our study, the γ-carboxyl groups of MTX were conjugated to the residual amino groups of the PEGylated CS-NPs. The drug-loading content of the (MTX + PEG)-CS-NPs was calculated as 7.23 ± 0.11%. The simple conjugation chemistry and appropriate drug-loading content could favor the dual-acting role of Janus-like MTX.

### In vitro stability tests

No significant variation of the particle size was observed in the (MTX + PEG)-CS-NPs even after incubation with PBS for a long period of time (Figure 4F). Notably, the CS-NPs (without PEGylation) could precipitate after 48 h in the presence of salts. It was implied that PEG could protect the (MTX + PEG)-CS-NP against ionic strength. No significant change of the particle size was also shown in the (MTX + PEG)-CS-NPs after incubation with 10% plasma for 120 h (Figure 4G). It should be inferred that PEG could reduce the plasma proteins adsorption, and more importantly, preserve the targeting potential of MTX. All of the results suggested that the (MTX + PEG)-CS-NPs were sufficiently stable to sustain physiological conditions for extended blood circulation.

### In vitro drug release profiles

**Figure 5:**
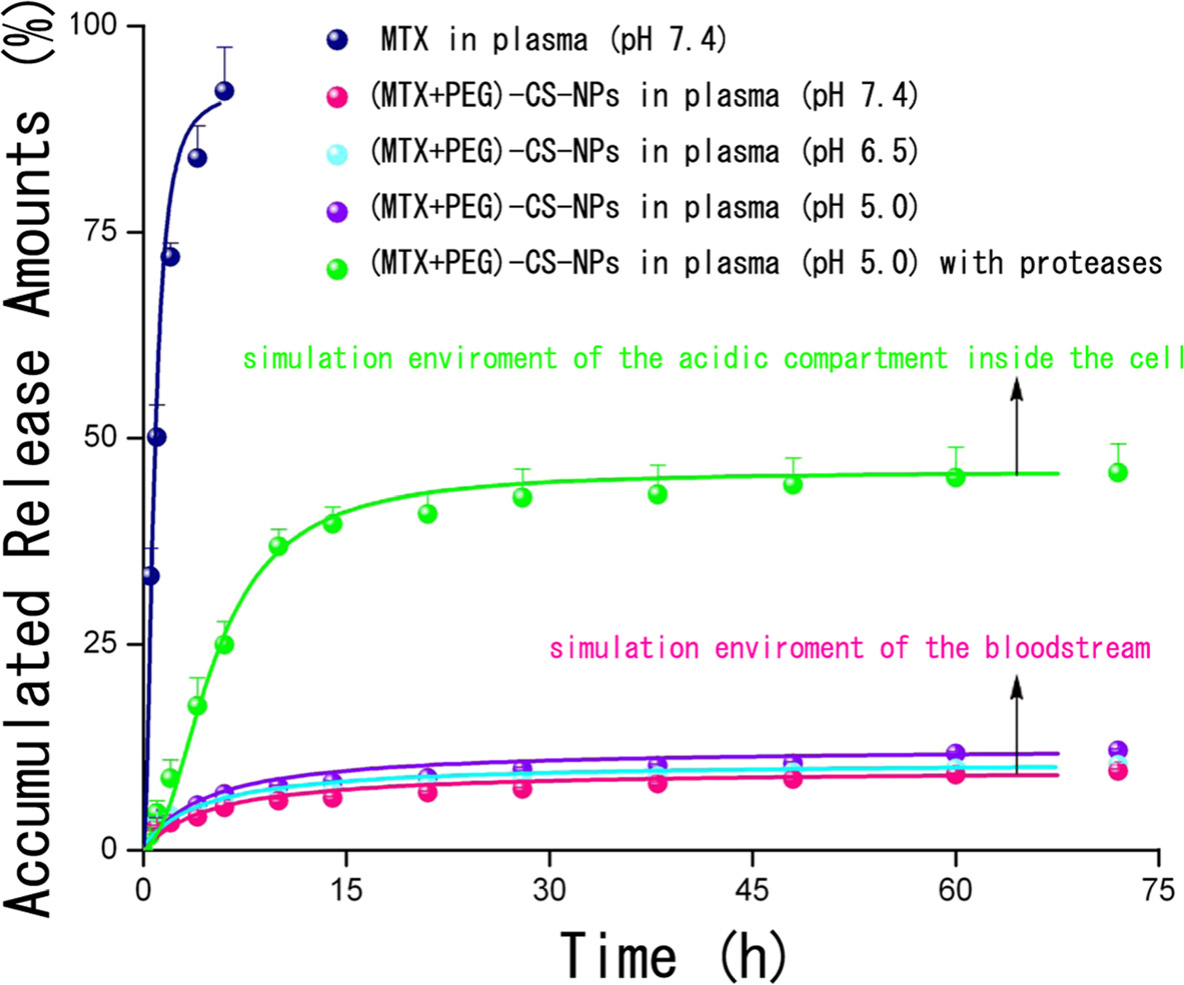
**In vitro drug release profiles of the (MTX + PEG)-CS-NPs in different physiological media (mean ± SD,*n*** **= 3).**

In vitro drug release profiles of the free MTX and (MTX + PEG)-CS-NPs were presented in Figure 5. To mimic the physiological conditions of the bloodstream, the (MTX + PEG)-CS-NPs were incubated with 10% plasma at pH 7.4. In sharp contrast to the free MTX with accumulated release amounts of almost 90% within 6 h, a more sustained release of the NPs was clearly observed due to the slow hydrolysis of amide bonds. Nevertheless, within 48 h, only no more than 10% of MTX from NPs was released at pH 7.4. Once intravenously administrated, the NPs could ensure minimal premature release of MTX during the circulation, and thereby greatly reduces the systemic toxicity. It was expected that the NPs will accumulate at the tumor site by the EPR effect. Once inside the tumor tissue, these MTX-targeted PEG-CS-NPs will be internalized by the tumor cells, largely via FA receptor-mediated endocytosis (discussed below).

It was well established that the amide bonds could be selectively cleaved at acidic pH by proteases (also called proteolytic enzymes) overexpressed in the tumor cells [33-36]. To simulate the physiological environment of the acidic compartments of the tumor cells, the (MTX + PEG)-CS-NPs were incubated with 10% plasma containing crude proteases at pH 5.0. MTX was released at a constant rate up to 10 h, reaching the accumulated release amounts more than 30%, we believed that proteases exerted a significant promotion effect to control drug release. As is reported, several kinds of particle-bound MTX attached by an amide linkage have been shown to be sensitive to the protease-mediated cleavage in the acidic environments, and hence, the lysosomal proteases could be responsible for the release of MTX from the particles [19,20,37,38]. Once the NPs were internalized by the target cells, the drug release could be significantly speeded up because of the long-lasting activity of proteases inside the cells, which can help to provide a sufficient intracellular level of MTX, and hence efficiently enhance the drug efficacy.

All of the results suggested that the covalent chemistry, preferring over physical adsorption, could be advantageous to preserve the targeting role of MTX. This could be of utmost importance, especially in vivo, where the avoidance of premature drug release and the untimely role change (from targeting to anticancer) of Janus-like MTX are pivotal.

### In vitro cellular uptake

**Figure 6:**
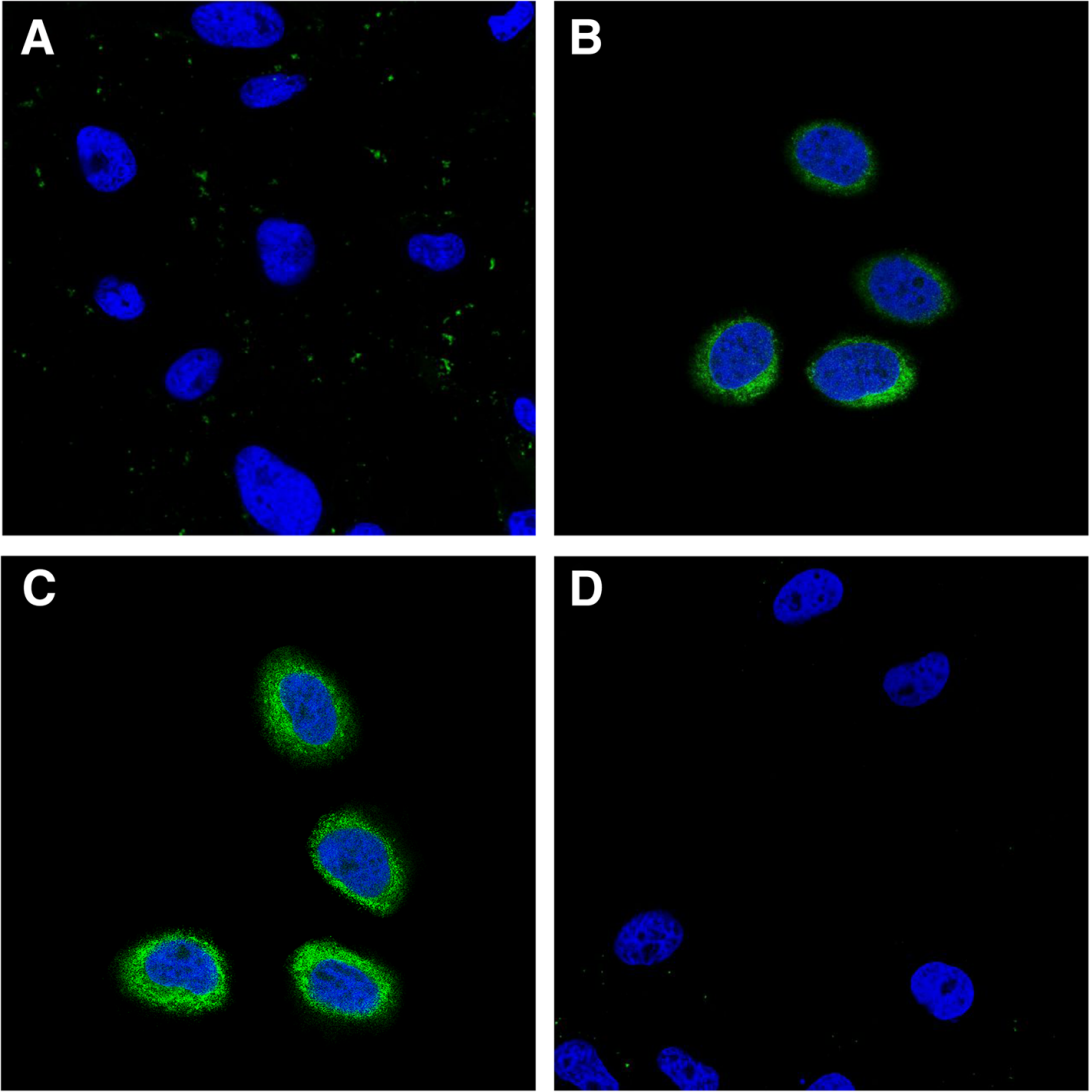
**In vitro cellular uptake of the (MTX + PEG)-CS-NPs.** Laser scanning confocal microscopy images of **(A)** HeLa cells incubated with the FITC-PEG-CS-NPs. **(B)** HeLa cells incubated with the FITC-(FA + PEG)-CS-NPs. **(C)** HeLa cells incubated with the FITC-(MTX + PEG)-CS-NPs. **(D)** HeLa cells blocked with excess of the free FA and then incubated with the FITC-(MTX + PEG)-CS-NPs. Incubation was carried out at 37°C for 6 h. The concentration of FITC was equivalent in all formulations. All images were taken using identical instrumental conditions and presented at the same intensity scale.

**Figure 7:**
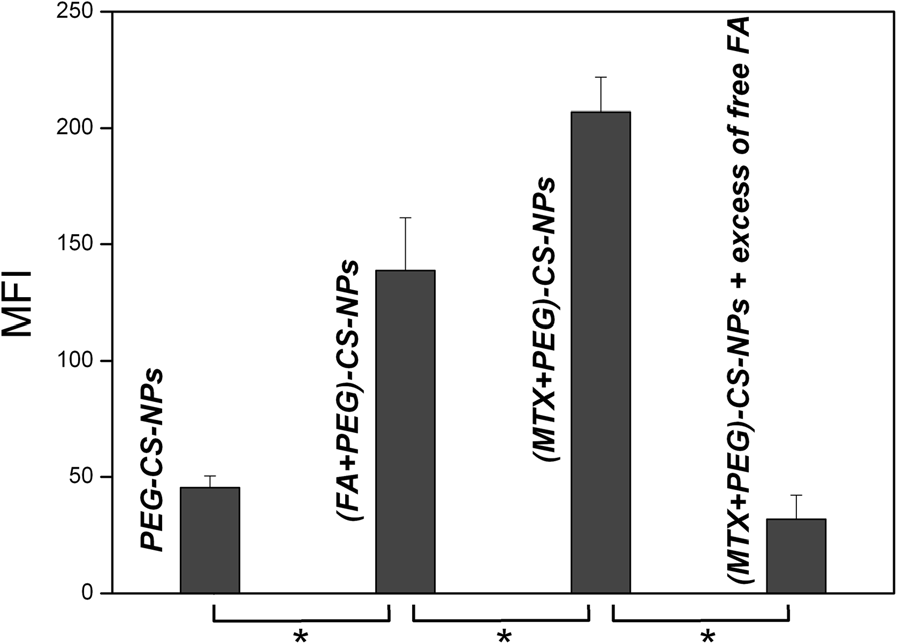
**Cellular uptake of FITC-PEG-CS-NPs, FITC-(FA + PEG)-CS-NPs, and FITC-(MTX + PEG)-CS-NPs (equivalent FITC concentration) on HeLa cells by flow cytometry (mean ± SD,*n*** **= 3).** Statistical significance: *P <0.05.

We investigated the comparative cellular uptake of different formulations by HeLa cells using laser scanning confocal microscopy (Figure 6). The FA modification enhanced the cellular uptake of the FITC-(FA + PEG)-CS-NPs compared with the FITC-PEG-CS-NPs (Figure 6A,B). These results can be explained by their distinct cellular uptake mechanisms. The FITC-PEG-CS-NPs might be taken up by the cells through nonspecific endocytosis, while the FA receptor-mediated endocytosis could further promote the cellular uptake of the FITC-(FA + PEG)-CS-NPs. More importantly, it was of interest to note that the MTX modification also significantly enhanced the cellular uptake of the FITC-(MTX + PEG)-CS-NPs (Figure 6C), indicating that MTX greatly improved the targeting effect. To evaluate the specificity of the cellular uptake of the FITC-(MTX + PEG)-CS-NPs, FA competition experiments were carried out. The internalization of the FITC-(MTX + PEG)-CS-NPs by the free FA-treated HeLa cells was greatly inhibited compared to the untreated HeLa cells (Figure 6D); these results suggested that the MTX functionalized nanoscaled drug delivery systems could specifically bind to FA receptor. But, equally important is that another possibility should not be neglected. Despite that, MTX has a suboptimal affinity for FA receptor compared with FA [13]; the multiple MTX ligands decorated on the (MTX + PEG)-CS-NPs can be bound simultaneously to multiple FA receptors overexpressed on the surface of cancer cells and promote the multivalent binding effect [39,40], which resulted in the collectively much tighter binding avidity [41,42] and progressively more efficient drug delivery through FA receptor-mediated internalization pathways that helped circumvent multiple-drug resistance (MDR) efflux mechanisms [43].We next made quantitative measurements of the cellular uptake of different PEG-CS-NPs formulations using flow cytometry. The mean fluorescence intensities (MFIs) of the cells after 4 h of incubation with different PEG-CS-NPs formulations were shown in Figure 7. The MFI should be directly correlated with the mean number of NPs taken up per cell. The MFI of HeLa cells treated with the FITC-(FA + PEG)-CS-NPs was significantly higher than the FITC-PEG-CS-NPs, and even the MFI of HeLa cells treated with the FITC-(MTX + PEG)-CS-NPs was also significantly higher than the FITC-(FA + PEG)-CS-NPs. These results also supported the idea of the targeting effect of both the FITC-(FA + PEG)-CS-NPs and FITC-(MTX + PEG)-CS-NPs to HeLa cells. The presence of excess of the free FA efficiently inhibited the cellular uptake of FITC-(MTX + PEG)-CS-NPs, which confirmed that the (MTX + PEG)-CS-NPs enter the cells through the FA receptor-mediated endocytosis.

These quantitative results were consistent with those qualitative results, giving a further proof of high targeting efficacy of the (MTX + PEG)-CS-NPs to HeLa cells. The possible reason is that the integral binding avidity of the (MTX + PEG)-CS-NPs towards FA receptor presents a great advantage of targeting efficacy outperformed that of the (FA + PEG)-CS-NPs towards FA receptor. As mentioned above, MTX has a suboptimal affinity to FA receptor compared with FA and may be less efficient to target to FA receptor than FA. Nevertheless, it was reported that multivalent binding avidity can be kinetically limited if the binding affinity of an individual receptor-ligand pair is too tight [44,45]. Well consistent with the above theoretical analysis, our result further suggested that the targeting specificity of the nanoscaled drug delivery systems for a particular cell type can be enhanced by the weaker binding affinity of each individual receptor-ligand pair. Indeed, the integral binding avidity plays a predominant role in the targeting efficacy; the higher integral binding avidity increases the targeting efficacy. Detailed in vivo targeting studies are necessary to further assess this possibility.

### In vitro cell viability studies

**Figure 8:**
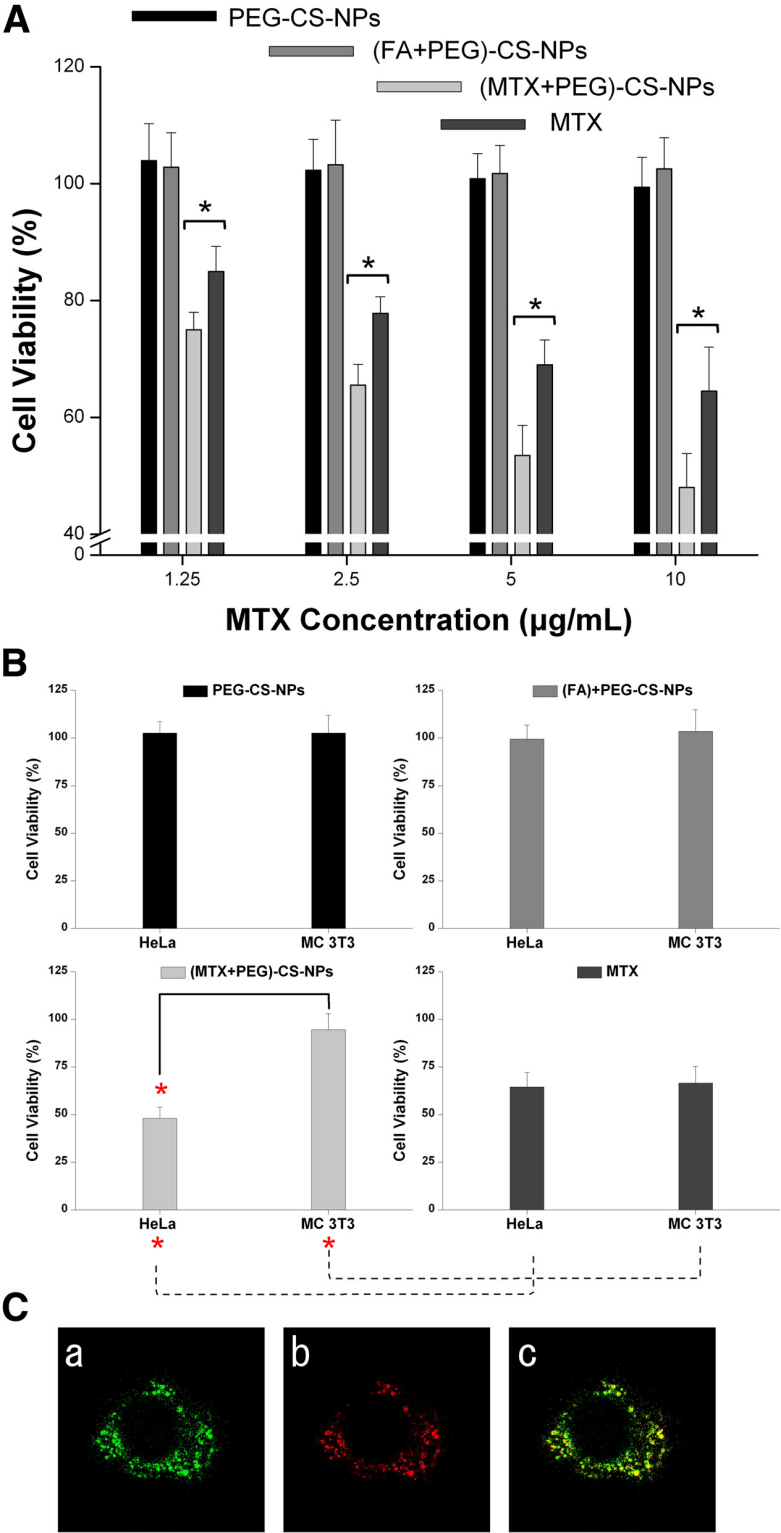
**In vitro cell viability and intracellular delivery. (A)** Cytotoxicity of the PEG-CS-NPs, (FA + PEG)-CS-NPs, (MTX + PEG)-CS-NPs, and free MTX against HeLa cells after 24 h of incubation (mean ± SD, *n* = 6). Statistical significance: *P < 0.05. **(B)** Cytotoxicity of the PEG-CS-NPs, (FA + PEG)-CS-NPs, (MTX + PEG)-CS-NPs, and free MTX at the highest MTX concentration (10 μg/mL) against HeLa cells (cancer cells) or MC 3 T3-E1 cell (normal cells) after 24 h of incubation (mean ± SD, *n* = 6). Statistical significance: *P < 0.05. **(C)** Intracellular delivery of the (MTX + PEG)-CS-NPs in HeLa cells after 4 h of incubation observed by laser scanning confocal microscopy. The late endosomes and lysosomes were stained by LysoTracker Red. (a) Green fluorescent FITC, (b) red fluorescent late endosomes/lysosomes, (c) overlay of (a) and (b).

The cytotoxicities of the PEG-CS-NPs, (FA + PEG)-CS-NPs, (MTX + PEG)-CS-NPs, and free MTX were assessed by MTT assays after incubation with HeLa cells for 24 h (Figure 8A). No visible cytotoxic effect of the PEG-CS-NPs was observed for HeLa cells, and the FA modification did not significantly alter the cytotoxic effect. In contrast, both the (MTX + PEG)-CS-NPs and free MTX exhibited a concentration-dependent cytotoxic effect towards HeLa cells. Moreover, delivering MTX by the (MTX + PEG)-CS-NPs significantly induced a much higher cytotoxicity compared to delivering the free MTX at the same drug concentration, even though this cell line is not MTX resistant. The result can be explained by the highly specific targeting efficiency, effectively sustained drug release, and efficient cytotoxicity enhancement effect of the MTX-targeted nanoscaled drug delivery systems, which lead to the enhanced cellular accumulation and retention of MTX.

The cytotoxicity of the (MTX + PEG)-CS-NPs (10 μg/mL) towards HeLa cells and MC 3 T3-E1 cells after 24 h of incubation was shown in Figure 8B. FA receptors were expressed at a high level on the surface of HeLa cells (cancer cells) but at a much lower level on MC 3 T3-E1 cells (normal cells). On the one hand, the cytotoxicity of the (MTX + PEG)-CS-NPs towards cancer cells was significantly higher compared to that of the free MTX. However, in the case of normal cells, the situation was opposite. On the other hand, the (MTX + PEG)-CS-NPs induced a marked cytotoxicity towards targeted cancer cells, but a slight cytotoxicity was observed for nontargeted normal cells, whereas the free drug affected both cell lines equally. The result indicated that the MTX modification played an important role in selectively enhanced cytotoxicity of the nanoscaled drug delivery systems [46]. All of these results also suggested that MTX was not prematurely released from the (MTX + PEG)-CS-NPs outside of HeLa cell, but was preferentially released inside HeLa cell after the cellular uptake of the (MTX + PEG)-CS-NPs.

To investigate the intrinsical mechanisms of the cytotoxicity of the (MTX + PEG)-CS-NPs, we investigated the subcellular localization of the FITC-labeled (MTX + PEG)-CS-NPs in HeLa cells by laser scanning confocal microscopy. As shown in Figure 8C, the internalized (MTX + PEG)-CS-NPs were found initially to be localized in the lysosomes, as evidenced by the yellow spots in the merged image obtained from the images of the (MTX + PEG)-CS-NPs (green) and late endosomes/lysosomes (red). The result indicated that the (MTX + PEG)-CS-NPs were internalized via the endocytosis pathway into the late endosomes/lysosome [47]. Indeed, after incubation for 4 h, some green fluorescent FITC-labeled (MTX + PEG)-CS-NPs were no longer located in the red fluorescent late endosomes/lysosomes, indicating the successful endo/lysosomal escape. In agreement with other reports [37,48], these results combined with the results of in vitro drug release and cell viability studies further proved that MTX was released from the (MTX + PEG)-CS-NPs inside the cells by the intracellular protease-mediated selective cleavage of peptide bond. These findings were also in agreement with other reports in the literature [49] that CS possessed the activity to some extent to escape the endo/lysosome.

## Conclusions

We presented the versatile, robust, and easy MTX-based PEGylated CS-NPs while validating MTX as a successful targeting ligand coordinated with a simple anticancer drug, and established the (MTX + PEG)-CS-NPs as a cocktail platform of specific targeting cooperated with enhanced anticancer activity. MTX was not prematurely released at off-target site but was intensively released at target site due to its sustained/protease-mediated drug release characteristic. To the best of our knowledge, the work for the first time explored the validation of Janus role of MTX based on the nanoscaled drug delivery system in vitro. Additionally, as MTX (a targeting ligand/a first drug) was introduced into one kind of drug carriers, one further advantage was that the drug delivery systems allowed the further introduction of a second ligand or a second drug for synergistic co-targeted delivery or synergistic co-delivery of drugs. Nevertheless, more details about in vivo targeting and anticancer investigations are indispensable to obtain a better understanding of the therapeutic effect of the (MTX + PEG)-CS-NPs, and relevant studies are in process.

## Competing interests

The authors declare that they have no competing interests.

## Authors’ contributions

FL and YL conceived and carried out the experiments, analysed the data, and wrote the paper. ZH designed the study, supervised the project, analysed the data, and wrote the paper. FY, MJ, and XY assisted in the synthesis and characterizations of the NPs. FC, HW, and JL assisted in the biological evaluations of the NPs. YL, ZH, and QZ provided insightful comments regarding the molecular mechanism. All authors read and approved the final manuscript.

## Authors’ information

Both authors FL and YL contributed equally and should be considered as co-first authors.
